# Evaluation of efficiency of controlled pollination based parentage analysis in a *Larix gmelinii* var. *principis-rupprechtii* Mayr. seed orchard

**DOI:** 10.1371/journal.pone.0176483

**Published:** 2017-04-27

**Authors:** Wenting Sun, Dade Yu, Mingliang Dong, Jian Zhao, Xiaoping Wang, Hongjing Zhang, Jinfeng Zhang

**Affiliations:** 1 National Engineering Laboratory for Tree Breeding, Key Laboratory of Genetics and Biotechnology Laboratory of State Forestry Administration, College of Biological Science and Biotechnology, Beijing Forestry University, Beijing, China; 2 Beijing Municipal Bureau of Landscape and Forestry, Beijing, China; 3 Hebei Forestry Research Institute, Shijiazhuang, China; Nanjing Forestry University, CHINA

## Abstract

Controlled pollination (CP) is an important tool for breeding programs to improve seed quality, as it rapidly generates desirable genotypes and maximizes genetic gains. However, few studies have evaluated the success rate of CP, especially in *Larix gmelinii* var. *principis-rupprechtii* Mayr. seed orchards. In this study, we estimated the rate of correct parentage in 257 CP progeny in an *L*. *gmelinii* var. *principis-rupprechtii* seed orchard from ten candidate parents using 13 microsatellites. The parentage exclusion probabilities of all combined loci in the single parent and parent pair tests were > 0.99, which was sufficient to distinguish the relatedness of the sampled individuals. Comparing the maximum likelihood-based parentage analysis results with breeding records revealed that the percentages of correctly identified maternal and paternal parents were 22.6% and 35.0% at 95% CL, respectively, suggestive of parent mislabeling and pollen contamination in the CP population. We conducted a pedigree reconstruction by identifying the expected parents and assigned maternity, paternity, and parent pairs to 176 (68.5%), 199 (77.4%), and 132 (51.4%) progeny, respectively. This study provides a reference for future selection of elite genotypes for commercial production. To increase the efficiency of CP, molecular markers should be used to correctly identify individuals in seed orchards before conducting CP.

## Introduction

Prince Rupprecht's larch (*Larix gmelinii* var. *principis-rupprechtii* Mayr.) is one of the most important native conifer species in northern China used for reforestation and to build non-food forests due to its growth speed, high timber quality, and numerous applications [1]. Natural populations are mainly distributed in Hebei and Shanxi provinces, China. Pinaceae species were first targeted for scientific breeding programs in China in the 1960s [2]. Currently, Prince Rupprecht's larch breeding is predominantly conducted in seed orchards to obtain elite genotypes. To produce high quality Prince Rupprecht's larch seeds, a seed orchard was built in Longtoushan, Hebei Province (41°56′N, 117°45′E) in 1976. It has an average altitude of 1386 m and comprises 100.2 ha of seed forest, which is the largest improved variety area of Prince Rupprecht's larch in the country. Seeds in the first- and second-generation seed orchards attained genetic gains up to 23 and 65%, respectively, through many tests, thereby improving modern varieties of Prince Rupprecht's larch [3].

Seed orchards are an essential component of improving genetic material for reforestation programs, including phenotypic selection of the base population, mating, testing, and genotypic selection of the breeding population, and producing improved seeds [4, 5, 6]. Optimizing the balance between effective genetic diversity and genetic gains in seed orchards is a primary objective for breeders. In general, breeders make decisions according to pedigree information to increase the heterosis effect, maintain high variability in progeny, and minimize inbreeding [7]. Blouin [8] used multiple analytical methods and conditioning likelihood using non-DNA information to reconstruct pedigrees. Funda et al. [9] estimated parental gametic contributions and the extent of pollen migration using a combination of pedigree reconstruction and a mating system analysis. Furthermore, paternity analyses of open- or controlled-pollination (CP) seed orchards have been reported in China for several tree species, including Korean pine (*Pinus koraiensis*) [10], Masson’s pine (*Pinus massoniana*) [11, 12], and *Pinus tabulaeformis* [13]. Knowing the parentage of progeny in seed orchards is important for understanding the exchange of genetic material among different generations and guiding the establishment of higher generation seed orchards.

Simple sequence repeats (SSRs) are a powerful tool in genetic analyses of forests due to their high information content, codominant nature, and wide genomic distribution [14, 15]. Molecular genetics-based parentage analyses have been conducted for some species. For example, 60 native Japanese chestnut (*Castenea crenata*) cultivars were estimated using hierarchical clustering and Bayesian model-based clustering to clarify the parentage and spread pattern of the cultivars [16]. The parentage of selectively bred cacao (*Theobroma cacao*) in Sulawesi, Indonesia was assessed to determine ancestry and genetic variability [17]. In addition, parentage was assigned to cacao clones from six seed gardens in Ghana to better understand their genetic relationships and increase the confidence in selecting parents for commercial plantations [18]. Emily et al. [19] reconstructed the parentage of shining gum eucalyptus (*Eucalyptus nitens*) from two New Zealand seed orchards using single nucleotide polymorphisms (SNPs) and SSR markers to compare the efficacy of the two DNA markers. The results suggest that, while microsatellite markers will remain a useful tool, SNPs will offer a new range of opportunities for high-throughput, genome-wide, or targeted genotyping.

Controlled pollination (CP) is essential to tree breeding programs to increase genetic gains and integrate targeted traits from individual genotypes [20]. Although only a few studies have confirmed the success of CP using SSR markers, including in loblolly pine (*Pinus taeda*) [21] and cacao [18], to the best of our knowledge, no reports have examined the efficiency of CP in Prince Rupprecht's larch seed orchards. In addition, parents mislabeling and pollen contamination are universal during germplasm collection and pollination, affecting the accuracy and efficiency of breeding programs [18, 22]. For example, in a cacao seed orchard, some individuals had been wrongly assigned to parental individuals, indicating that mislabeling existed before the parents had been planted [18]. Most studies on Prince Rupprecht's larch have been related to genetic diversity and population structure [23, 24]. Although one study conducted a parentage analysis of open pollination in a Prince Rupprecht's larch seed orchard [2], no studies have evaluated parentage in CP populations using SSR markers.

In this study, the parentage of 267 individuals, including 257 CP progeny and ten candidate parents, sampled from a first-generation seed orchard were estimated using a set of SSR markers. The aims of this study were: (1) to estimate the rate of correct CP by comparing breeding records of the expected parents and identify the underlying sources of error in the breeding program to propose methods to reduce such errors during the production of genetically improved seeds; and (2) To use the microsatellite data to estimate the genetic parameters for parentage determination and individual identification and conduct a pedigree reconstruction of the CP *L*. *gmelinii* var. *principis-rupprechtii* seed orchard. Pedigree reconstructions can provide a practical tool for guiding the future selection of superior individuals and parents to produce improved progeny.

## Material and methods

### Plant materials and DNA extraction

All plant material was collected in May 2012 from the national key seed base of *L*. *gmelinii* var. *principis-rupprechtii* in Longtoushan, Hebei Province, China. In total, 267 individuals were selected for this study, including ten parents sampled from the first-generation seed orchard and 257 progeny generated from seven and five elite parents that were used as mothers and fathers, respectively via controlled crossing (Table 1). Healthy young leaves were collected from each tree, frozen in liquid nitrogen, and stored at –80°C in an ultra-low-temperature freezer until the genomic DNA extraction. Genomic DNA was extracted from leaf tissue using a modified cetyltrimethylammonium bromide method [25]. DNA quality was evaluated using 0.8% agarose gel electrophoresis. DNA concentration was determined using a NanoDrop 2000 spectrophotometer and diluted to a concentration of 50 ng/μL for the subsequent microsatellite amplification.

**Table 1:** Candidate parents and number of their sampled controlled-pollinated progeny from an *L*. *gmelinii* var. *principis-rupprechtii* seed orchard.

Maternal Parent Code	Paternal Parent Code					
	205#	98#	53#	77#	120#	∑
43#	9	10	9	10	8	46
49#	0	9	0	0	9	18
53#	10	8	0	0	3	21
55#	10	7	9	10	10	46
56#	10	7	8	10	9	44
59#	10	10	9	8	7	44
77#	10	10	8	0	10	38
∑	59	61	43	38	56	257

### Microsatellite amplification

DNA was genotyped using 13 SSR markers, which included seven SSR markers obtained from the transcriptome sequencing data of *Larix gmelinii* var. *principis-rupprechtii* and six expressed sequence tag (EST)-SSR markers developed from ESTs of Pinaceae from the National Center for Biotechnology Information (NCBI) database (Table 2) [24, 26]. The microsatellite simplex PCR amplification was conducted with a reaction volume of 25 μL containing 100 ng of genomic DNA, 200 μmol dNTP, 0.25 μmol of each primer pair, 1U of Taq DNA polymerase with 1× buffer containing Mg^2+^. The amplification was performed under the following conditions: initial denaturation for 5 min at 94°C, 29 cycles of 30 s at 94°C, 30 s at the optimized annealing temperature, elongation for 45 s at 72°C, and a final extension for 5 min at 72°C. The products were separated on a non-denaturing 8% polyacrylamide gel using the HT-SCZ04 high flux vertical electrophoresis tank and electrophoresed at 130 V for 1.5 h. The fragments were visualized with 0.1% silver nitrate. Alleles were visually compared with a 100-bp DNA ladder as a standard to determine their size.

**Table 2:** Information on the 13 SSR primers.

Primer	Primer sequence (5′–3′)	Size (bp)	Repeat motif	Melting temperature (°C)
F1*	F AAGGAGGAGGGTCAGGGAA	160–190	(TCAGGC)5	55
	R CATGCGGAGGTTGAGTTG			
F11*	F AATGAAGCCAACTGTGCC	200–220	(ATT)4+(TGT)4	55
	R ATTCACCCCAATCCCATC		+(GTGGCA)4	
F17*	F CGGCTGAGGTTGCGAAAGA	140–180	(AGTCC)4	56
	R CAATTACATAAGTGGGACGAG		+(GTCCA)6	
F42*	F AACACTTTAATCCCCTCCC	190–220	(CAGGAA)5	51
	R CTGGACCTGATACTCCTCTTC			
F94*	F GCCGTTGACAACAATTACAT	150–180	(ACTGG)5	55
	R AAAGAATAGCAACCCGCAGT			
F97*	F GTGGATTTGTTGACAGGGAG	190–210	(AT)8	55
	R GTCGTTGTAATGAGGGTGGA			
D15	F GCGAAGATACGGTGAGCAA	180–200	(GA)6	55
	R TCCGAGAAGGACGCCATTA			
D42	F TACTCTTGTCGCCGCTATCTAC	230–250	(TA)8	55
	R GGTCCTACCACCAAACTTCACT			
D63	F CCCCTCGTATTCGCTCAAGT	220–240	(CAG)6	55
	R CAGCCCAGTTCCATTTTCGT			
D77	F CGGGAACTCACGGAAAAG	260–280	(CTG)7	55
	R CAACCGAATCATCCATACTACA			
DN4	F TATGTTTCCTTTTCCGAT	200–220	(TGAGC)5	53
	R TCTTCCTCCTTCATTGCT			
DN7	F ATGGCATCGCAGAATCAGT	170–200	(GGTCCA)7	55
	R TTTGGTGGCGGTGGTAAT			
D111	F TCACCTCTTGTTCCTCGTC	190–210	(GCT)7	55
	R ATCATTCTCGCCATACTCG			

Primers labeled with * were from Fan Yingming et al. 2014, while unlabeled primers were from Dong Mingliang et al.2016.

### Data analysis

The genetic polymorphism parameters of each SSR locus were calculated with POPGENE ver. 1.32 software [27]. The parameters included the number of alleles (N_a_) and observed and expected heterozygosity (H_o_, H_e_). The polymorphism information content (PIC) of each primer pair was calculated with PIC_CAL ver. 0.6 software [26]. The parentage analysis, individual identification of all samples, and parentage exclusion probabilities were estimated with Cervus ver. 3.0.7 software [28]. The null allele frequency was estimated with an individual inbreeding model-based estimator [29]. The Hardy-Weinberg equilibrium of each locus was tested to characterize the microsatellite markers. The simulation parameters were: 300000, 500000 and 100000 parent-offspring pairs in the analysis of maternities, paternities and parent pairs, respectively, 7 candidate maternal parents and 5 candidate paternal parents (i.e., number of parents according to the breeding records), 99% of candidate parents were selected, and 98% of the loci were typed. The proportion of loci mistyped was calculated as $\sum \left(2q_{i}\left(1-q_{i}\right)+q_{i}^{2}\right)/L$, where *q*_*i*_ was the null allele frequency at the *i*th locus among 267 trees, and *L* was the number of loci. The error rate over the 13 loci was estimated to be 2.8%. [30] For each parent-offspring pair, the critical likelihood value (LOD score) was obtained from simulations at either an 80% (relaxed) or 95% (strict) confidence level (CL).

## Results

### Microsatellite marker polymorphisms

In total, we genotyped 267 individuals from 13 SSR markers, with data missing for 37 of 3,738 data points (0.8%). The N_a_ of all samples ranged from 2 to 6, with a mean N_a_ of 3.9. The mean H_o_ and H_e_ values were 0.503 and 0.492, respectively. The mean H_e_ was higher than that of the mean H_o_ for five primer pairs (D15, D63, D77, F17, and DN4), indicating a lack of heterozygotes at these loci. D42 and D63 had the highest (0.612) and lowest (0.189) PICs, respectively (Table 3). The alleles of two loci (D63 and F97) were over-concentrated (PIC < 0.25), while four primer pairs had high degrees of polymorphism (PIC > 0.5) and the others had intermediate levels of polymorphism (0.25 < PIC < 0.5). High genetic diversity resulted in a high statistical power for the parentage analysis when combining all markers. In addition, the total parentage exclusion probability for the single parent and parent pair tests was > 0.99, indicating that the set of markers used for this analysis had good identification ability and was adequate for determining parentage and recognizing individuals. Among the 13 primers pairs, the null allele frequency was < 0.05, considered to be a meaningful threshold [31]. One marker (D42) deviated from Hardy-Weinberg equilibrium, which may have resulted from the presence of null alleles. Overall, the genotyped population demonstrated strong consistency with the expected genotypic proportions under Hardy-Weinberg equilibrium.

**Table 3:** Summary of the genetic informativeness of 13 microsatellite markers in *Larix gmelinii* var. *principis-rupprechtii*.

Locus	N_a_	H_o_	H_e_	PIC	P_first_	P_second_	P_pair_	HW	F_Null_
D111	4	0.537	0.521	0.426	0.139	0.241	0.365	NS	–0.0127
D15	3	0.502	0.532	0.466	0.141	0.275	0.419	NS	0.0189
D42	5	0.707	0.675	0.612	0.249	0.408	0.579	***	–0.0442
D63	3	0.184	0.199	0.189	0.019	0.099	0.18	NS	0.0245
D77	6	0.583	0.637	0.594	0.219	0.39	0.576	NS	0.0162
DN4	2	0.481	0.501	0.375	0.125	0.187	0.281	NS	0.0151
DN7	5	0.613	0.6	0.545	0.193	0.35	0.52	NS	–0.0013
F1	5	0.518	0.481	0.455	0.127	0.289	0.466	NS	–0.0394
F11	2	0.555	0.459	0.353	0.104	0.176	0.267	NS	–0.0832
F17	4	0.478	0.485	0.43	0.121	0.254	0.4	NS	0.0086
F42	5	0.662	0.627	0.58	0.227	0.397	0.582	NS	–0.0241
F94	4	0.461	0.441	0.397	0.102	0.235	0.379	NS	–0.0088
F97	3	0.263	0.244	0.222	0.03	0.116	0.2	NS	–0.0458
Average	3.9	0.503	0.492	0.434	0.138	0.263	0.401	-	-
Combined all loci	-	-	-	-	0.9967	0.9990	0.9994		-

The analysis was constructed using 257 progeny from 10 parents; N_a_, number of alleles; H_o_, observed heterozygosity; H_e_, expected heterozygosity; PIC, polymorphism information content; P_first_, exclusion probability of the first parent; P_second_, exclusion probability of the second parent; P_pair_, exclusion probability of the parent pair; HW, significance of deviation from Hardy-Weinberg equilibrium; NS, not significant; F_Null_, estimated null allele frequency.

*** p < 0.001 (significant)

### Controlled pollination success

**Fig 1:**
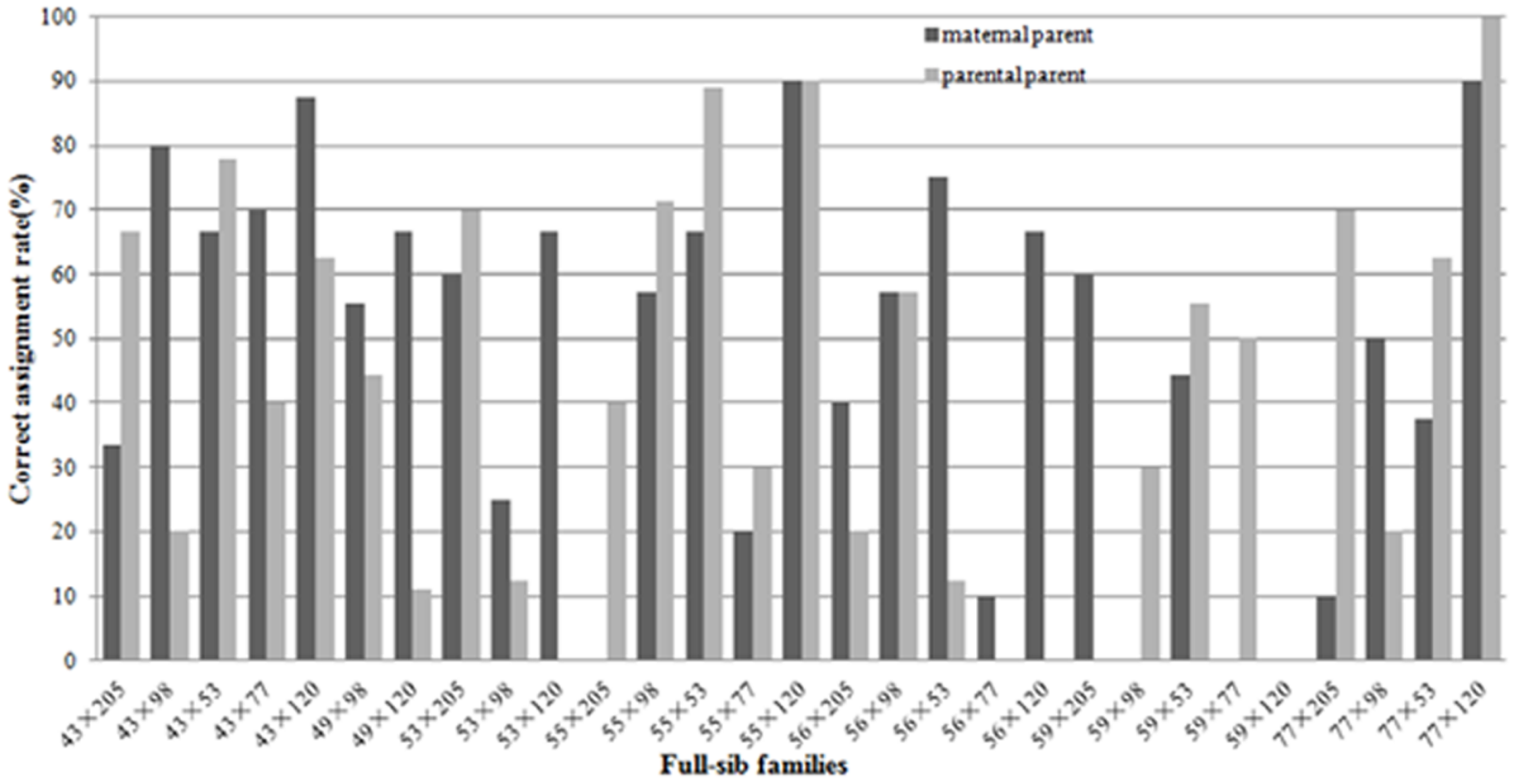
Correct maternity and paternity assignment rate of 257 control-pollinated individuals in 29 full-sib families in a *Larix gmelinii* var. *principis-rupprechtii* seed orchard.

To estimate the efficacy of CP, the most likely maternal and paternal parents of the 257 offspring were identified from ten candidate parents. Compared with the breeding records, 58 of 257 progeny had been assigned the correct mother in the breeding records; similarly, 90 of 257 progeny had been assigned the correct father at 95% CL. However, the family of which all progeny had been completely correctly matched with the mother was zero (Table 4). In addition, the rate of correctly assigned maternal parents was ≤ 50% in 12 families (Fig 1), of which only one ramet had the correct mother in 53×205, 53×120, 55×98, 55×77, 56×205, 56×120, 77×205 and 77×120 at the 95% CL. Moreover, 53×98, 55×205, 59×98, 59×77, 59×120, 77×98, 77×53 had no maternity matches (Table 4). The analysis revealed that some of the parental ramets in mother-reproduced progeny might have been mislabeled before hybridization. Based on the paternal parent analysis, all families exhibited pollen contamination. At the 95% CL, no progeny of families 49×120, 53×120, 56×77, 56×120 and 59×205 had been assigned to the correct paternal parent, indicating that they had a contamination rate of 100%. In three families (56×205, 56×53 and 59×120), only one progeny had the correct paternal parent, representing contamination rates of 90%, 87.5% and 85.7%, respectively (Table 4). Clone 77 was the main source of contamination in 56×53, accounting for 75% of the contamination rate. Meanwhile, clones 53, 98 and 120 were the source of contamination in 56×77, 56×120 and 59×205 (Table 4). In some families (e.g., 43×53 and 43×77), no expected paternity was observed, suggestive of contamination from external sources of pollen (Table 4). Overall, 43×53, 43×120, 53×205, 55×53, 55×120 and 77×120 had the highest rates of assigned parent pairs (> 60%), while 53×98, 55×77, 56×77, 59×98 and 59×120 had the lowest rates (< 30%) (Fig 1). The rate of correct maternal assignment (47.9%) was nearly equal with that of paternal assignment (43.5%), indicating that pollen contamination was common with mislabeled parents in this CP population.

**Table 4:** Success rate of CP in 257 progeny from 29 full-sib families in a *Larix gmelinii* var. *principis-rupprechtii* seed orchard.

Family	N	Maternal parent			Paternal parent		
		NC-f	NC-f-S	Contaminant parent^※^	NC-m	NC-m-S	Contaminant parent^※^
43×205	9	3	3	-	6	6	120(1)
43×98	10	8	4	55(1)	2	2	77(3), 120(2)
43×53	9	6	2	55(2)	7	5	-
43×77	10	7	5	-	4	2	-
43×120	8	7	4	-	5	3	-
49×98	9	5	3	-	4	4	-
49×120	9	6	2	-	1	0	-
53×205	10	6	1	56(1), 43(1)	7	5	77(1)
53×98	8	2	0	59(2)	3	3	77(1)
53×120	3	2	1	-	0	0	77(2)
55×205	10	0	0	59(2)	4	4	-
55×98	7	4	1	-	5	4	-
55×53	9	6	4	49(1),77(1)	8	7	98(1)
55×77	10	2	1	49(2)	3	2	-
55×120	10	9	6	-	9	6	-
56×205	10	4	1	49(1), 77(1)	2	1	120(6)
56×98	7	4	3	43(1)	4	3	120(1)
56×53	8	6	4	-	1	1	77(6)
56×77	10	2	2	43(1)	0	0	53(5), 120(3)
56×120	9	6	1	-	0	0	53(5), 98(4)
59×205	10	6	4	55(3)	0	0	53(3), 120(4)
59×98	10	0	0	-	3	3	-
59×53	9	4	4	49(1),56(1)	5	5	205(1),77(2)
59×77	8	0	0	49(1)	4	3	-
59×120	7	0	0	-	1	1	205(2)
77×205	10	1	1	-	7	5	-
77×98	10	5	0	55(1)	2	2	53(3)
77×53	8	3	0	49(1)	5	4	-
77×120	10	9	1	-	10	9	-
Total	257	123(47.9%)	58(22.6%)	-	112(43.5%)	90(35.0%)	-

NC-f-S and NC-m-S represent the number of offspring with the correct maternal parent and paternal parent at the 95% CL respectively; N, number of offspring sampled per family; NC-f, number of offspring with the correct maternal parent at the 80% CL; NC-m, number of offspring with the correct paternal parent at the 80% CL.

^※^ The number in the parenthesis is the amount of contamination observed for a particular parent.

### Pedigree reconstruction

To reconfirm the parentage of the 257 individuals to the 10 candidate parents, we used SSR markers to assign maternity, paternity, and the most likely parent pair. Overall, maternity was assigned to any single parent of the 10 parents for 176 of the 257 progenies (68.5%), while the paternity of 199 progenies (77.4%) was successfully assigned to any single parent at 80% confidence levels (Table 5). The LOD scores were slightly lower for the paternity assignment, likely explaining the large proportion of unassigned individuals in this data array. At the 95% CL, paternity (54.5%) was assigned to a greater percentage of progeny than maternity (23.3%) (Table 5).

**Table 5:** Assessment of parentage for 257 CP individuals from 29 full-sib families in a *Larix gmelinii* var. *principis-rupprechtii* seed orchard.

Parameter	Maternity assignment	Paternity assignment	Full parentage assignment
Number of offspring	257	257	257
Total number (percentage) of progeny assigned to a single parent or parent pair	176(68.5%)	199(77.4%)	132 (51.4%)
Critical LOD score of parentage identification at the 95% CL	2.70	1.82	4.75
Critical LOD score of parentage identification at the 80% CL	0.21	0.04	1.45
Number (percentage) of progeny assigned to a single parent or parent pair at the 95% CL	60 (23.3%)	140 (54.5%)	58 (22.6%)
Number (percentage) of progeny assigned to a single parent or parent pair at the 80% CL	116(45.1%)	59(22.9%)	74 (28.8%)
Number (percentage) of unassigned progeny	81 (31.5%)	58 (22.6%)	125(48.6%)

Higher LOD scores were obtained in the assignment of most likely parent pairs of the 257 progeny. In total, 51.4% of all progeny (132 of 257) were assigned any parent pair, including 58 (22.6%) at the 95% CL (Table 5). However, 125out of 257 progeny were unassigned, suggestive of clonal ramet mislabeling or pollen contamination. Based on the maternity, paternity, and full parentage assignments, we conducted a pedigree reconstruction of the CP progeny in the seed orchard (S1 Table). When the most likely mother (or father) assigned in the tests was the father (or mother) of the cross combination, we selected the second most likely option as the mother or father; if the LOD score of the second most likely parent was below the standard (i.e., the LOD score of the maternity and paternity assignment was < 0.21 and 0.04, respectively), we determined that no parent from the seed orchard could be identified, possibly due to the mislabeling of parents or pollen contamination from other parents than the ten candidate parents.

## Discussion

### The estimates of probabilities for parentage assignments

Polymorphic microsatellites were a useful and inexpensive tool for assigning parentage in Prince Rupprecht's larch. The H_o_ and H_e_ of 13 SSR markers were within the same ranges as natural populations [24], although the mean H_e_ (0.492) was higher than that of other Prince Rupprecht's larch populations [26, 2, 24, 32] (Table 3). The results revealed that the polymorphism of these microsatellites had sufficient statistical power to identify individuals and assign parentage. The exclusion power of genetic markers is one of the most important elements of parentage assignments. The number of markers must change with the number of sampled individuals to achieve a high exclusion probability of the correct parents [33]. The multi-locus combined parentage exclusion probabilities obtained in this study were > 0.99 for the single parent and parent pair tests (Table 3). Similar estimates were reported in a parentage assignment study of a loblolly pine seed orchard; the estimated of probabilities were 0.99 for the single parent test and > 0.9999 for the parent pair test [21]. To our knowledge, this is the first report to estimate the parentage exclusion probability using SSR markers in Prince Rupprecht's larch. The high exclusion probabilities obtained in this study suggest that SSR markers are sufficiently powerful to precisely identify the parents of CP progeny in Prince Rupprecht's larch seed orchards. Although SNPs are increasingly popular in genetic studies due to their higher rates of correct genotyping [34], SSR markers will remain an easily accessible, informative, and cheap option for molecular analyses of Prince Rupprecht's larch and similar breeding programs.

### Causes of incorrect parentage

The maternity and paternity assignment analyses had error rates of 52.1% and 56.5% at 80% CL (Table 4), respectively, suggesting that CP in this seed orchard had been affected by various sources of error. Some progeny with a maternal parent in disagreement with the breeding records may have been generated from other individuals from the same orchard or other seed gardens. During the breeding process, careful collection of seeds from different clone ramets is important to guarantee the correct mother. When seeds are accidentally mixed with those from different parents and misidentified, maternity errors occur due to the mismatching of ramets [21]. This labeling error has been reported in other studies. For example, in a loblolly pine seed orchard, mislabeling occurred mostly among parents that had undergone mass CP, which influenced the accuracy of maternity in the breeding program [21]. In addition, two forms of mislabeling, homonymous and synonymous mislabeling, were identified in a cacao seed garden [18]. Paternity errors generally occur during pollination due to the introduction of external pollen from neighboring gardens [35]. In this study, paternal contamination was the result of both pollen from other parents within the seed orchard and external sources of pollen (Table 4). Pollen contamination is a serious problem that can decrease the genetic quality of an orchard’s crops [36]. In a maritime pine (*Pinus pinaster* Ait.) polycross seed orchard, the minimum pollen contamination rate was 36%, resulting in expected genetic gain losses of 18–50% [37]. Moreover, a maritime pine clonal seed orchard had an alien pollen contamination rate of 52.4%, determined via nuclear microsatellite analysis [36]. Similarly, a Scots pine (*Pinus sylvestris* L.) seed orchard had an estimated pollen contamination of 52%, which impacted the expected genetic gain to such a degree that no selection gain was observed [38].

### Pedigree reconstruction

Pedigree reconstructions of seed orchards are an effective method of providing genetic information to assign parentage, estimate parental reproductive success, and evaluate the efficacy of management strategies [39, 5]. Although we successfully identified paternity and maternity in 77.4%and 68.5% of progeny, respectively, using 13 SSR markers, some progeny could not be assigned to any of the ten parents from this seed orchard. Estimating the maternal or paternal parents of these unassigned progenies would be complex and time consuming, since the candidate parents could be any ramets of any clone from this or other seed orchards. To eliminate this error, breeding programs should verify the parentage of individuals using molecular markers before breeding. However, the parentage assignments in this study can be used to select parents of elite individuals for further breeding and provide a method for ascertaining the accuracy of breeding materials to conduct successful genetic studies in the future. Another study conducted a full and partial reconstruction pedigree of lodgepole pines (*Pinus contorta*) and found that the full pedigree reconstruction was superior to the partial pedigree reconstruction, enabling the estimation of both paternal- and maternal-related fertility parameters [39]. In Douglas fir (*Pseudotsuga menziesii*), pedigree reconstruction was used to identify the maternal and paternal parents of each seed and estimate the clonal reproductive success and selfing rate and determine the proportion of seeds sired by outside pollen sources [40]. Similarly, Funda et al. compared the reproductive success of three seed orchards [lodgepole pine, Douglas fir, and western larch (*Larix occidentalis*)] based on pedigree reconstructions [41]. These studies show that pedigree reconstruction is an important component of genetic studies of seed orchards, particularly for parentage assignments and reproductive success estimations.

## Conclusions

We used microsatellite markers to evaluate the success rate of controlled crossing and identify the parentage of 257 CP progeny in an *L*. *gmelinii* var. *principis-rupprechtii* seed orchard. The results revealed that maternal and paternal parents were correctly identified for only slightly more than half of all progeny, possibly due to ramet mislabeling and pollen contamination, including pollen from this seed orchard and alien pollen from other orchards. Therefore, we advocate the use of DNA markers which have a sufficiently high statistical power to identify individuals using several microsatellites to appraise and correctly identify all clonal ramets. Before conducting CP, the maternity and paternity of each ramet should be confirmed to eliminate factors that could affect the success of controlled crossing. Moreover, the pedigree reconstruction of the 257 CP progeny provides a strong basis for recommending elite individuals for the breeding program and guiding parentage selection to improve *L*. *gmelinii* var. *principis-rupprechtii*.

## Supporting information

S1 Table Details for pedigree reconstruction. (DOC)
