# Supplementary material for: Evaluation of efficiency of controlled pollination based parentage analysis in a Larix gmelinii var. principis-rupprechtii Mayr. seed orchard
Source: PLoS One. 2017 Apr 27;12(4):e0176483. doi: 10.1371/journal.pone.0176483 (PMC5407790; doi:10.1371/journal.pone.0176483)
Supplement: S1 Table — (DOC) [file pone.0176483.s001.doc]

Supplementary Table S1 Pedigree reconstruction of 257 CP progeny from 29 families in a *Larix gmelinii* var. *principis-rupprechtii* seed orchard.

| Family | Ramet | F | M |  | Family | Ramet | F | M |  | Family | Ramet | F | M |
| --- | --- | --- | --- | --- | --- | --- | --- | --- | --- | --- | --- | --- | --- |
| 43×205 | 1 | 43 | - |  | 43×120 | 1 | 43 | 120 |  | 53×98 | 1 | ***77*** | - |
|  | 2 | - | 205 |  |  | 3 | 43 | 120 |  |  | 2 | - | 98 |
|  | 3 | 43 | 205 |  |  | 4 | - | 120 |  |  | 3 | 53 | ***205*** |
|  | 4 | - | 205 |  |  | 5 | 43 | - |  |  | 4 | - | 98 |
|  | 5 | 43 | 205 |  |  | 6 | 43 | - |  |  | 5 | ***59*** | 98 |
|  | 6 | - | 205 |  |  | 7 | 43 | ***-*** |  |  | 6 | 53 | 98 |
|  | 7 | 43 | ***120*** |  |  | 8 | 43 | 120 |  |  | 7 | ***59*** | 98 |
|  | 8 | - | 205 |  |  | 9 | 43 | 120 |  |  | 8 | - | - |
|  | 9 | 43 | 205 |  | 49×98 | 1 | 49 | ***-*** |  | 53×120 | 1 | ***-*** | ***77*** |
| 43×98 | 1 | - | - |  |  | 2 | 49 | - |  |  | 2 | 53 | ***77*** |
|  | 2 | 43 | - |  |  | 3 | 49 | 98 |  |  | 3 | 53 | ***77*** |
|  | 3 | 43 | ***77*** |  |  | 4 | - | 98 |  | 55×205 | 1 | - | - |
|  | 4 | 43 | 98 |  |  | 5 | 49 | 98 |  |  | 2 | - | 205 |
|  | 5 | 43 | 98 |  |  | 6 | 49 | - |  |  | 3 | - | 205 |
|  | 6 | 43 | ***77*** |  |  | 7 | - | - |  |  | 4 | - | 205 |
|  | 7 | ***55*** | ***120*** |  |  | 8 | - | 98 |  |  | 5 | - | - |
|  | 8 | 43 | ***120*** |  |  | 9 | 49 | ***-*** |  |  | 6 | - | - |
|  | 9 | 43 | - |  | 49×120 | 1 | 49 | ***98*** |  |  | 7 | - | 205 |
|  | 10 | 43 | ***77*** |  |  | 2 | 49 | ***-*** |  |  | 8 | - | - |
| 43×53 | 1 | ***-*** | ***77*** |  |  | 3 | 49 | ***98*** |  |  | 9 | - | - |
|  | 2 | ***55*** | 53 |  |  | 4 | 49 | - |  |  | 10 | ***59*** | ***-*** |
|  | 3 | ***53*** | 53 |  |  | 5 | 49 | 120 |  | 55×98 | 1 | 55 | - |
|  | 4 | 43 | 53 |  |  | 6 | 49 | 120 |  |  | 2 | - | 98 |
|  | 5 | 43 | 53 |  |  | 7 | 49 | ***98*** |  |  | 3 | - | 98 |
|  | 6 | 43 | 53 |  |  | 8 | - | ***98*** |  |  | 4 | 55 | 98 |
|  | 7 | 43 | 53 |  |  | 9 | - | ***98*** |  |  | 5 | 55 | ***53*** |
|  | 8 | 43 | 53 |  | 53×205 | 1 | ***56*** | - |  |  | 6 | - | 98 |
|  | 9 | 43 | ***-*** |  |  | 2 | 53 | 205 |  |  | 7 | 55 | 98 |
| 43×77 | 1 | 43 | 77 |  |  | 3 | ***43*** | ***77*** |  | 55×77 | 1 | - | 77 |
|  | 2 | 43 | 77 |  |  | 4 | - | 205 |  |  | 2 | - | 77 |
|  | 3 | ***-*** | ***120*** |  |  | 5 | 53 | - |  |  | 3 | - | - |
|  | 4 | 43 | - |  |  | 6 | 53 | 205 |  |  | 4 | ***49*** | - |
|  | 5 | 43 | - |  |  | 7 | 53 | 205 |  |  | 5 | ***-*** | 77 |
|  | 6 | 43 | - |  |  | 8 | 53 | 205 |  |  | 6 | ***49*** | - |
|  | 7 | 43 | 77 |  |  | 9 | - | 205 |  |  | 7 | ***-*** | ***53*** |
|  | 8 | 43 | 77 |  |  | 10 | 53 | 205 |  |  | 8 | ***49*** | ***53*** |
|  | 9 | - | - |  |  |  |  |  |  |  | 9 | 55 | - |
|  | 10 | 43 | 77 |  |  |  |  |  |  |  | 10 | 55 | 77 |

F, expected maternity; M, expected paternity; Italicized values indicate that the expected parents were mismatched in the breeding records; Un-italicized values indicate that the expected parents matched the breeding records.

Supplementary Table S1 (continued)

| Family | Ramet | F | M |  | Family | Ramet | F | M |  | Family | Ramet | F | M |
| --- | --- | --- | --- | --- | --- | --- | --- | --- | --- | --- | --- | --- | --- |
| 55×53 | 1 | - | ***98*** |  | 56×53 | 1 | ***43*** | 53 |  | 59×98 | 1 | ***-*** | ***53*** |
|  | 2 | 55 | 53 |  |  | 2 | 56 | ***205*** |  |  | 2 | - | - |
|  | 3 | ***77*** | 53 |  |  | 3 | 56 | ***77*** |  |  | 3 | - | - |
|  | 4 | 55 | 53 |  |  | 4 | 56 | ***77*** |  |  | 4 | - | 98 |
|  | 5 | 55 | 53 |  |  | 5 | ***77*** | ***77*** |  |  | 5 | - | - |
|  | 7 | ***49*** | 53 |  |  | 6 | 56 | ***77*** |  |  | 6 | - | 98 |
|  | 8 | 55 | 53 |  |  | 7 | 56 | ***77*** |  |  | 7 | ***-*** | - |
|  | 9 | 55 | 53 |  |  | 8 | 56 | ***77*** |  |  | 8 | - | ***-*** |
|  | 10 | 55 | 53 |  | 56×77 | 1 | 49 | ***98*** |  |  | 9 | - | - |
| 56×98 | 1 | ***43*** | ***77*** |  |  | 2 | 56 | ***53*** |  |  | 10 | - | 98 |
|  | 2 | 56 | 98 |  |  | 3 | 56 | ***53*** |  | 59×53 | 1 | 59 | 53 |
|  | 3 | 56 | 98 |  |  | 4 | - | ***120*** |  |  | 2 | 59 | 53 |
|  | 4 | 56 | 98 |  |  | 5 | 56 | ***53*** |  |  | 3 | 59 | 53 |
|  | 5 | - | ***120*** |  |  | 6 | 56 | ***53*** |  |  | 4 | 59 | 53 |
|  | 6 | - | 98 |  |  | 7 | 56 | ***53*** |  |  | 5 | ***49*** | 53 |
|  | 7 | 56 | 98 |  |  | 8 | ***43*** | ***120*** |  |  | 6 | 59 | 53 |
| 55×120 | 1 | 55 | 120 |  |  | 9 | 56 | ***120*** |  |  | 7 | - | ***205*** |
|  | 2 | - | ***-*** |  |  | 10 | 56 | ***120*** |  |  | 8 | ***77*** | ***-*** |
|  | 3 | 55 | 120 |  | 56×120 | 1 | ***59*** | ***53*** |  |  | 9 | ***56*** | ***77*** |
|  | 4 | 55 | 120 |  |  | 2 | 56 | ***53*** |  | 77×98 | 1 | 77 | ***53*** |
|  | 5 | 55 | 120 |  |  | 3 | - | ***98*** |  |  | 2 | 77 | - |
|  | 6 | 55 | 120 |  |  | 4 | 56 | ***53*** |  |  | 3 | 77 | ***53*** |
|  | 7 | 55 | 120 |  |  | 5 | 56 | ***98*** |  |  | 4 | 77 | - |
|  | 8 | 55 | 120 |  |  | 6 | 56 | ***53*** |  |  | 5 | - | - |
|  | 9 | 55 | 120 |  |  | 7 | - | ***98*** |  |  | 6 | - | 98 |
|  | 10 | 55 | 120 |  |  | 8 | 56 | ***53*** |  |  | 7 | - | 98 |
| 56×205 | 1 | - | ***120*** |  |  | 9 | 56 | ***98*** |  |  | 8 | ***55*** | ***53*** |
|  | 2 | 56 | - |  | 59×205 | 1 | 59 | ***-*** |  |  | 9 | - | 98 |
|  | 3 | ***49*** | ***120*** |  |  | 2 | 59 | ***-*** |  |  | 10 | 77 | - |
|  | 4 | 56 | 205 |  |  | 3 | ***55*** | ***53*** |  | 59×77 | 1 | - | - |
|  | 5 | - | ***120*** |  |  | 4 | 59 | ***-*** |  |  | 2 | - | 77 |
|  | 6 | ***49*** | - |  |  | 5 | ***55*** | ***53*** |  |  | 3 | ***49*** | 77 |
|  | 7 | 56 | 205 |  |  | 6 | ***55*** | ***53*** |  |  | 4 | ***-*** | 77 |
|  | 8 | - | ***120*** |  |  | 7 | - | ***120*** |  |  | 5 | - | - |
|  | 9 | 56 | ***120*** |  |  | 8 | 59 | ***120*** |  |  | 6 | ***49*** | 77 |
|  | 10 | ***77*** | ***120*** |  |  | 9 | 59 | ***120*** |  |  | 7 | 59 | 77 |
|  |  |  |  |  |  | 10 | 59 | ***120*** |  |  | 9 | - | 77 |

F, expected maternity; M, expected paternity; Italicized values indicate that the expected parents were mismatched in the breeding records; Un-italicized values indicate that the expected parents matched the breeding records.

Supplementary Table S1 (continued)

| Family | Ramet | | F | | M | |  | Family | | Ramet | | | F | | M | |  | Family | | Ramet | | | F | M |
| --- | --- | --- | --- | --- | --- | --- | --- | --- | --- | --- | --- | --- | --- | --- | --- | --- | --- | --- | --- | --- | --- | --- | --- | --- |
| 59×120 | 1 | - | | - | |  | | | 77×205 | | 1 | 77 | | ***-*** | |  | | | 77×120 | | 1 | - | | 120 |
|  | 2 | - | | ***98*** | |  | | |  | | 2 | - | | 205 | |  | | |  | | 2 | 77 | | 120 |
|  | 3 | - | | ***-*** | |  | | |  | | 3 | - | | 205 | |  | | |  | | 3 | 77 | | 120 |
|  | 4 | - | | ***205*** | |  | | |  | | 4 | - | | 205 | |  | | |  | | 4 | 77 | | 120 |
|  | 5 | - | | ***205*** | |  | | |  | | 5 | - | | 205 | |  | | |  | | 5 | 77 | | 120 |
|  | 6 | - | | - | |  | | |  | | 6 | - | | 205 | |  | | |  | | 6 | 77 | | 120 |
|  | 7 | - | | - | |  | | |  | | 7 | - | | 205 | |  | | |  | | 7 | 77 | | 120 |
| 77×53 | 1 | ***-*** | | 53 | |  | | |  | | 8 | - | | 205 | |  | | |  | | 8 | 77 | | 120 |
|  | 2 | 77 | | 53 | |  | | |  | | 9 | - | | 205 | |  | | |  | | 9 | 77 | | 120 |
|  | 3 | 77 | | 53 | |  | | |  | | 10 | 77 | | 205 | |  | | |  | | 10 | 77 | | 120 |
|  | 4 | - | | ***-*** | |  | | |  | |  |  | |  | |  | | |  | |  |  | |  |
|  | 5 | - | | 53 | |  | | |  | |  |  | |  | |  | | |  | |  |  | |  |
|  | 6 | ***49*** | | 53 | |  | | |  | |  |  | |  | |  | | |  | |  |  | |  |
|  | 7 | 77 | | - | |  | | |  | |  |  | |  | |  | | |  | |  |  | |  |
|  | 8 | - | | ***-*** | |  | | |  | |  |  | |  | |  | | |  | |  |  | |  |

F, expected maternity; M, expected paternity; Italicized values indicate that the expected parents were mismatched in the breeding records; Un-italicized values indicate that the expected parents matched the breeding records.
